# Prevention of Diabetes in the NOD Mouse by Intra-muscular Injection of Recombinant Adeno-associated Virus Containing the Preproinsulin II Gene

**DOI:** 10.1155/EDR.2001.129

**Published:** 2001

**Authors:** Rahul M. Jindal, M. Karanam, Rita Shah

**Affiliations:** 1 Department of Surgery Indiana University School of Medicine Indianapolis USA; 2 Department of Surgery University of Glasgow Glasgow G11 6NT UK

## Abstract

Using the Adeno-associated virus (AAV) as a gene delivery vehicle, we have constructed a recombinant vector containing the full length rat preproinsulin gene (vLP-1). Utilizing the well described non-obese diabetic (NOD) mouse model, an experimental group (n=10) of animals were intramuscularly (IM) injected with 10^7^ rAAV virions containing the insulin gene and compared to a mock-injected control group (n=10). Blood glucose (glc) was then measured weekly for 16 weeks. Data showed that the experimental group contained 70% euglycemic animals (defined as glc <200mg/dL) * versus* 10% of the control animals (P<.05) at 14 weeks. Mean weight in the treated group was greater than the untreated group. Insulin mRNA was detected at the injection site of all of the treated animals, but not controls. Complete destruction of islets was confirmed by histology ruling out the possibility of spontaneous reversal of insulinitis. We conclude that IM delivery of the insulin gene in the NOD mouse was able to prevent clinical DM up to 14 weeks in a majority of treated animals. Our experimental data suggests that gene therapy may be an alternative treatment for IDDM in the future.


Int. Jnl. Experimental Diab. Res., Vol. 2, pp. 129–138
Reprints available directly from the publisher
Photocopying permitted by license only

© 2001 OPA (Overseas Publishers Association) N.V.
Published by license under
the Harwood Academic Publishers imprint,
part of Gordon and Breach Publishing,
a member of the Taylor & Francis Group.
Printed in the U.S.A.

### Prevention of Diabetes in the NOD Mouse by Intra-muscular Injection of Recombinant Adeno-associated Virus Containing the Preproinsulin II Gene

RAHUL M. JINDAL[a, b,*], M. KARANAM[b] and RITA SHAH[a]

[a]*Department of Surgery, Indiana University School of Medicine, Indianapolis, USA;* [b]*Department of Surgery, University of Glasgow, Glasgow, UK-G11 6NT*

*(Received 17 January 2001; Revised 14 April 2001; In final form 29 May 2001)*

**Using the Adeno-associated virus (AAV) as a gene delivery vehicle, we have constructed a recombinant vector containing the full length rat preproinsulin gene (vLP-1). Utilizing the well described non-obese diabetic (NOD) mouse model, an experimental group (n = 10) of animals were intramuscularly (IM) injected with $10^7$ rAAV virions containing the insulin gene and compared to a mock-injected control group (n = 10). Blood glucose (glc) was then measured weekly for 16 weeks. Data showed that the experimental group contained 70% euglycemic animals (defined as glc < 200 mg/dL) *versus* 10% of the control animals (P < .05) at 14 weeks. Mean weight in the treated group was greater than the untreated group. Insulin mRNA was detected at the injection site of all of the treated animals, but not controls. Complete destruction of islets was confirmed by histology ruling out the possibility of spontaneous reversal of insulinitis. We conclude that IM delivery of the insulin gene in the NOD mouse was able to prevent clinical DM up to 14 weeks in a majority of treated animals. Our experimental data suggests that gene therapy may be an alternative treatment for IDDM in the future.**

*Keywords*: Gene therapy; Diabetes; NOD mouse; Recombinant adeno-associated virus

*Abbreviations*: Ad2: adenovirus 2; DM: Diabetes mellitus; rAAV: recombinant adeno-associated virus; Glc: Glucose; IDDM: Insulin dependent diabetes mellitus; IM: Intramuscular; moi: multiplicity of infection; NOD: Non-obese diabetic; pAAV\AD: helper plasmid; RSV-LTR: Rous sarcoma virus long terminal repeat; $rI_2$: rat preproinsulin II gene

### INTRODUCTION

Diabetes Mellitus (DM) in humans is the result of either lack of insulin (Type I) or insulin resistance (Type II). The impact of DM on morbidity and mortality is considerable. Since the discovery of insulin, DM is considered a manageable disease, however, secondary complications of DM are considerable and lead to significant morbidity and mortality. Alternative strategies for insulin delivery and the long-term treatment for type I DM are being actively investigated. Implantable insulin pumps have been tried, but technical complications and cost are prohibitive. Clinical trials using insulin as a prophylactic agent to delay the onset of DM in patients with genetic susceptibility are underway.[1] Pancreatic islet cell transplants have had mixed results;[2] although the results of whole organ pancreas transplants have improved in the last few years, the severe shortage of cadaver organs will be a limiting factor along with additional problems

*Corresponding author. e-mail: r.jindalr@clinmed.gla.ac.uk associated with life-long immunosuppression. The prospects of xenotransplantation of islets appears to have serious immunological, ethical and infectious problems.

We report here a novel mechanism for insulin delivery *in vivo*. It was generally assumed that gene therapy would be the dominant treatment for genetic diseases by the mid- to late-90's. However, considerable drawbacks involving immunogenicity of the delivery systems and the multifactorial nature of most diseases proved a difficult hurdle to overcome. Since the middle 90's, new vector delivery systems have emerged that have allowed more efficient gene delivery with improved results in the short-term. In particular, the recombinant adeno-associated virus (rAAV) appears to be a significant advance in non-immunogenic gene delivery system. In addition to an absence of local immune response, the rAAV is unique because it establishes a latent, stable infection with integration in a site-specific manner. Recent applications have involved HIV, Cystic Fibrosis, Fanconi Anemia, Interleukin-2, GMCSF production and alteration of drug resistance.[3] Furthermore, IM gene transfer has become the route of choice due to accessibility, vascularity, and large tissue mass.[4]

### MATERIALS AND METHODS

### Construction of the Recombinant rAAV

The steps in the construction of recombinant rAAV containing the rat preproinsulin II gene ($rI_2$) gene have been described previously.[5] Briefly, the pLP-1 plasmid incorporating the $rI_2$ gene was engineered by releasing the $rI_2$ gene with a RSV-LTR promoter from pBC I2BI (ATCC), purified, and inserted into the BamH1 site of rAAV vector plasmid pWP-19. The recombinant plasmid (pLP-1), together with pAAV\AD helper plasmid, was co-transfected into human kidney carcinoma cell line 293 (ATCC). The rAAV genome was rescued using helper adenovirus and packaged into mature AAV virions designated vLP-1. The recombinant AAV vector stocks were subsequently purified on cesium chloride equilibrium density gradients followed by DNase I digestion and heat inactivation at 56°C to render free of any original plasmid DNA and adenovirus.

### Animals

For studies reported herein, we used 11 week old NOD mice at initiation of the experiments; 60–80% untreated female animals develop diabetes by approximately 12 weeks. Infectious virions ($1.7 \times 10^9$) were given IM (right thigh) to 10 NOD mice; controls ($n = 10$) were injected with saline.

### End Point

Animals were sacrificed at week 16 and tissues examined for the presence of $rI_2$ gene as described below. Tissues were also examined for pathological changes by histology.

### PCR Methods

Animals were sacrificed to determine expression of $rI_2$ mRNA. Injection site muscle, muscle from non-injected site (opposite limb), liver, and were obtained and placed into Trizol-BRL (Life Technologies, Inc.). Total RNA (~1 ug) extracted from the individual tissues was subjected to RT-PCR using Superscript™ RT (Gibco, BRL). Primers used for $rI_2$ amplification were 5′-GCTACAGTCGGAAACCATC-3′ and 5′-ACAGGGTAGTGGTGGGCCT-3′, and oligo-dT (Oligo's Etc., Wilsonville, Oregon). For beta-actin amplification the following primers were used: 5′-CCTGACCCTGAAGTACCCCA-3′ AND 5′-CGTCAGGCAGCTCATAGCTC-3′. Reverse transcription was performed according manufacturer's directions (Superscript™ RT, Gibco, BRL). PCR was then carried out using standard methods.[6] The amplified products were electrophoresed on 1.5% agarose gels, transferred to nylon membranes (Boeringer

Mannheim, Indianapolis) and analyzed by Southern analysis using [$\alpha$ $^{32}$P]-labeled probe ($rI_2$ or beta-actin). After hybridization, membranes were washed and exposed to X-omat film (Eastman Kodak) using standard methods.[6]

### Measurement of Blood glc

Blood glc (analyzed by glucometer, Boehringer Mannheim, Inc.) was determined on day 1, 3, and at weekly intervals.

### Histology

Pancreata were examined histologically for the presence of insulitis. Other mice tissues were examined for evidence of cellular infiltration by H&E stain.

### Insulin RIA

Serum was harvested from animals at 1 week, and at the end of the study. A standard amount of serum was analyzed using a rat insulin RIA kit per manufacturers recommendations (Linco, St. Louis, MO). Insulin was compared between responder experimental animals and non-responder experimental animals *versus* controls. The cross-reactivity if this assay with proinsulin is estimated to be 70% (Linco does not have purified rat proinsulin-personal communication, Marcia Niss, Linco, St Louis, MO).

### RESULTS

In the present study, we investigated the effect of IM injection on rAAV containing the $rI_2$ into 11 week old female NOD mice. Experimental and control animals were randomly divided into 2 groups of 10 animals each. Animals were then injected IM with vLP-l or culture media and blood glc recorded weekly at 3 PM. Animals in the injected group had statistically significant lower random blood glc levels than controls (Fig. 1A). Re-evaluation of the raw data using arbitrary euglycemia cut-offs of 140 mg/dL (Fig. 1B) and 200 mg/dL (Fig. 1C) showed that the majority of experimental animals maintained glc levels below the cutoff (70% at 14 weeks *vs*. 10% of the controls).

In addition to improved glc levels, vLP-1-injected animals had a higher mean weight (Fig. 2), and appeared visibly more healthy than controls (Fig. 3). At 16 weeks, the animals

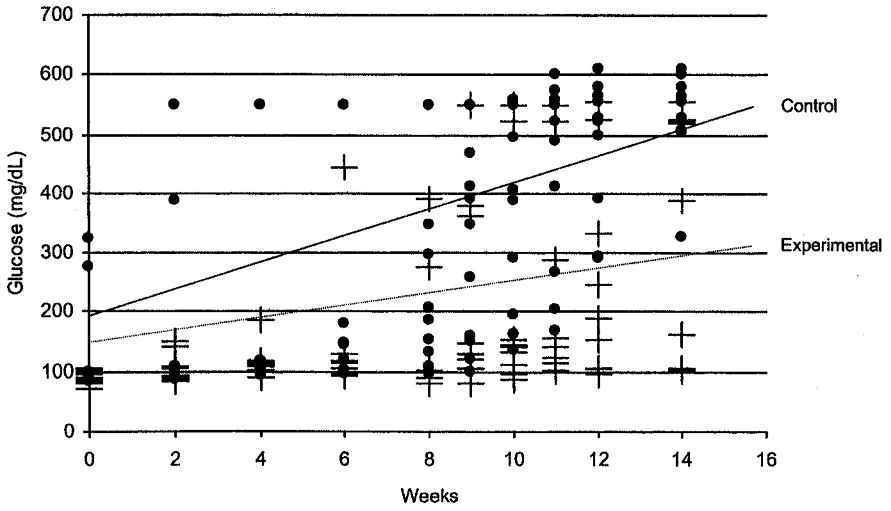

FIGURE 1A Individual glucose levels for all animals. All glucose levels were measured at the same time weekly. + denoted the glucose level in experimental animals while • denotes the glucose level in control animals.

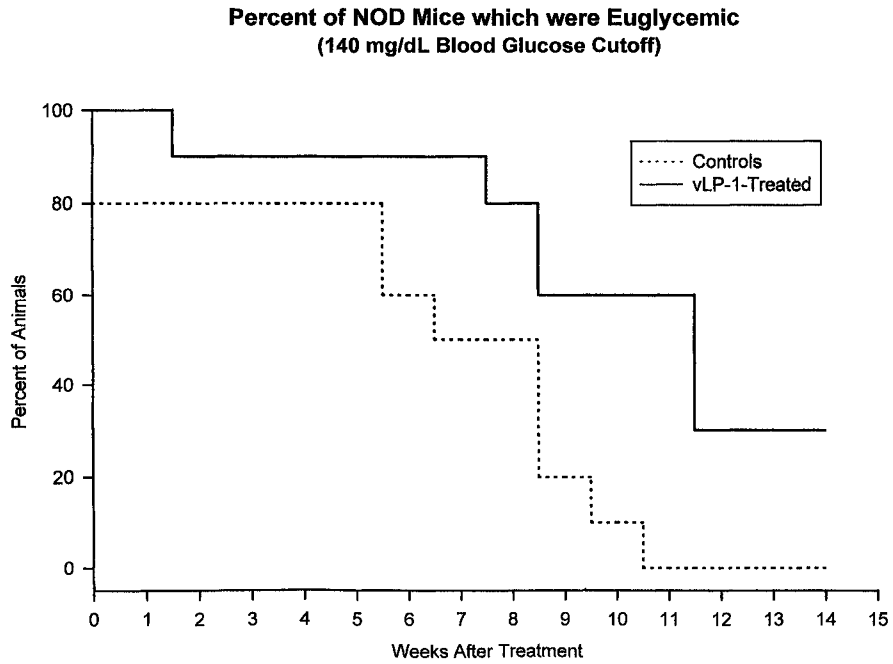

FIGURE 1B Kaplan-Meier curve showing percent of euglycemic animals as defined by an arbitrary glucose cutoff of 140mg/dL.

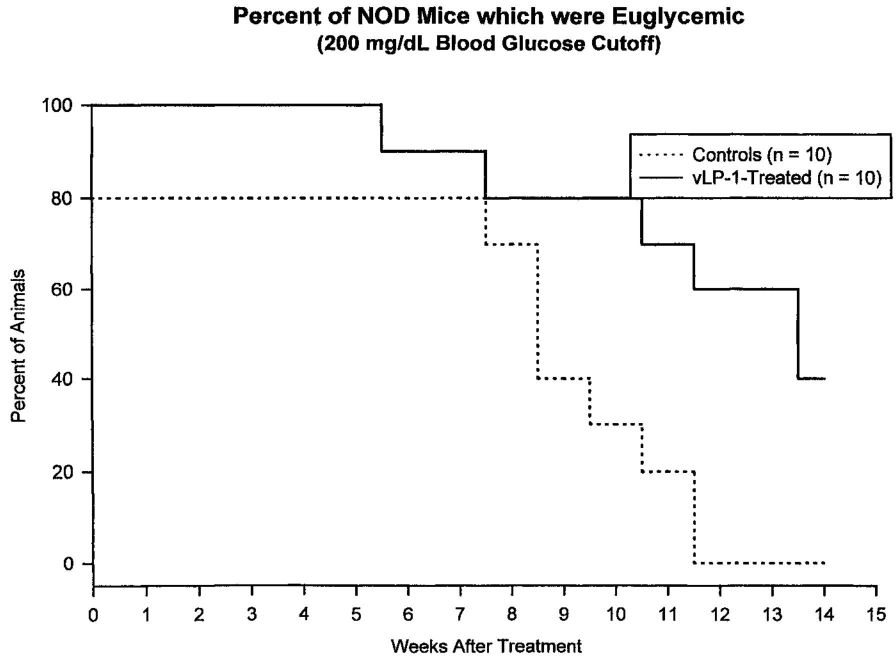

FIGURE 1C Kaplan-Meier curve showing percent of euglycemic animals as defined by an arbitrary glucose cutoff of 200mg/dL.

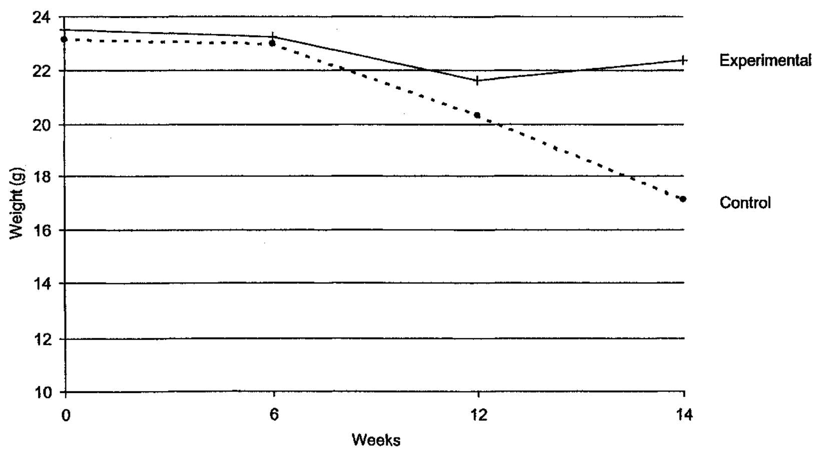

FIGURE 2 Mean weight of experimental (n = 10) and control (n = 10) animals. Weights were measured at the same time glucose levels were obtained.

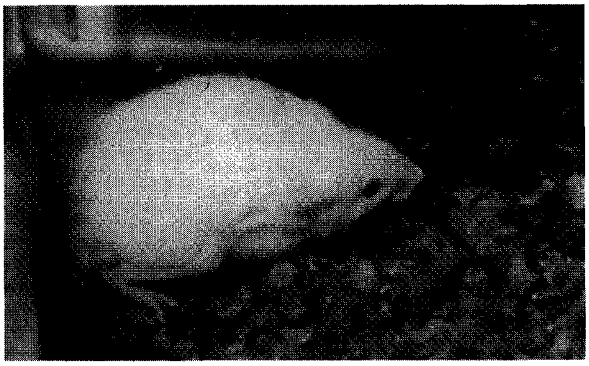

Control

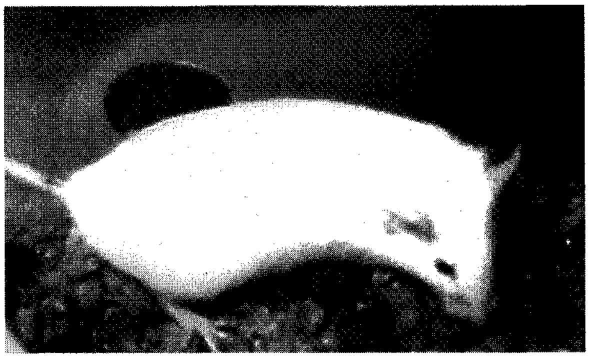

Experimental

FIGURE 3 Photograph of a representative experimental and control animal at 14 weeks.

were sacrificed and various tissues were examined by hematoxylin and eosin (H&E) staining. The degree of insulinitis in control and experimental animals was found to be equal (Fig. 4A). In addition, there was no evidence of immune infiltrate at the injection site or in other tissues (Fig. 4B). We stained islets of rAAV injected animals with anti-insulin antibody and found that insulin producing beta cells were not seen in these islets (Fig. 4C). Figure 4D represents control for insulin staining. Finally, examination of the injection site, as well as liver and spleen for extra-pancreatic rat mRNA by RT-PCR consistently identified insulin mRNA in the muscle injection site only in the non-diabetic animals. Animals that had received vLP-I but became diabetic did not express insulin mRNA and were used as an internal control. Figure 5A shows a representative experimental animal expressing rat insulin mRNA from the injection site (lane 6) but not in other tissues. Tissues from a diabetic experimental animal did not show any insulin mRNA expression including at the injection site (Lanes 1–3). Beta-actin RT-PCR was performed on the same samples to ensure the presence of intact mRNA (Fig. 5B). Control

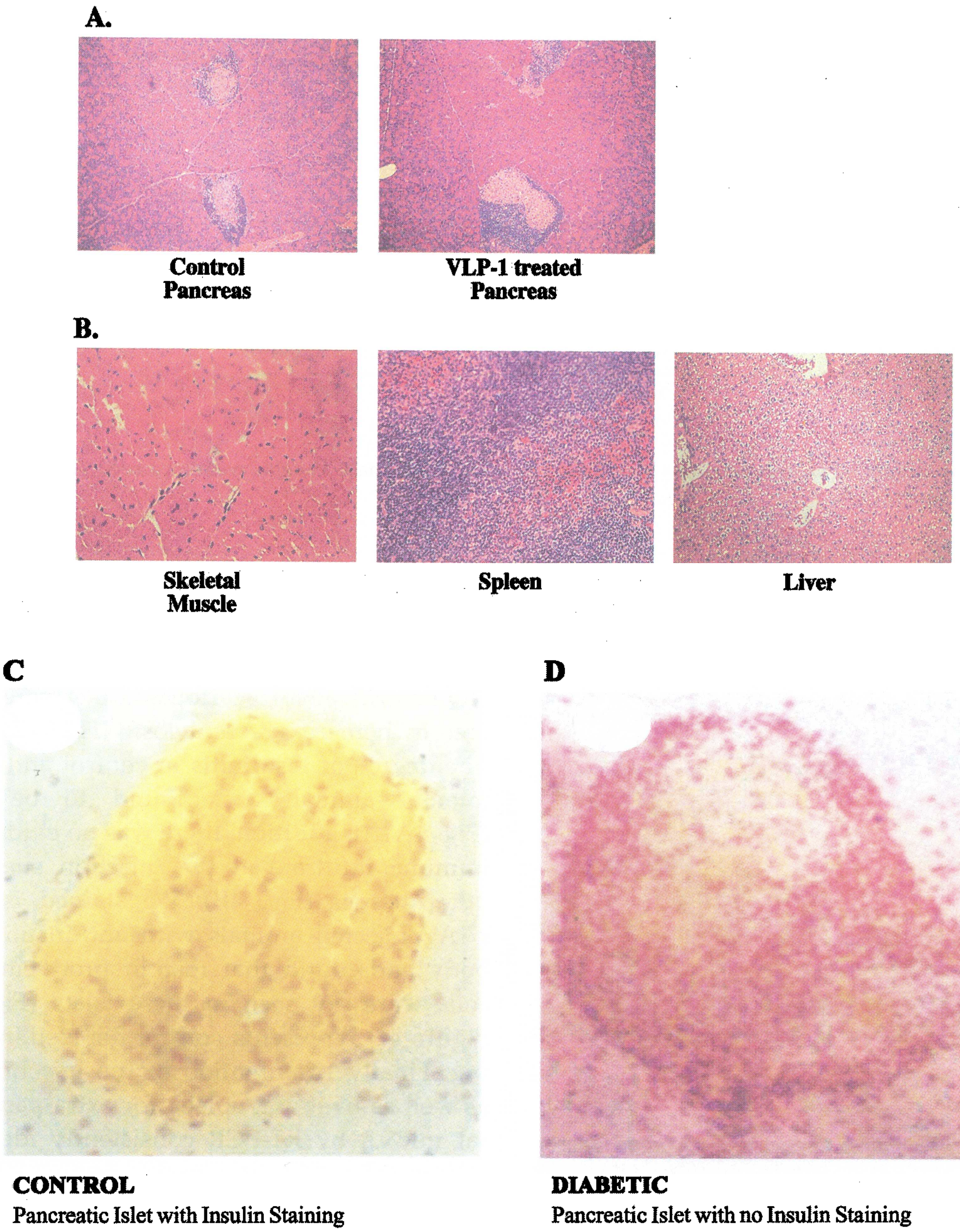

FIGURE 4 (A) H&E staining of the representative experimental and control animal pancreata showing no difference in the presence of insulinitis. (B) Representative tissues of an experimental animal showing no immune infiltration at any organ site including the injected muscle. (C) Pancreas obtained from normoglycaemic NOD mice at 16 weeks as control, showing normal staining of pancreas islets. (D) Pancreas obtained from NOD mouse at 16 weeks showing absence of insulin stained islets (stained with anti-insulin antibody).

non-injected animals did not express rat insulin mRNA (data not shown). The level of circulating insulin was examined using a rat insulin RIA. Despite the presence of overt insulinitis as determined by histology, animals with normal blood sugars at 16 weeks had higher levels of insulin than diabetic experimental animals and diabetic controls (Fig. 6).

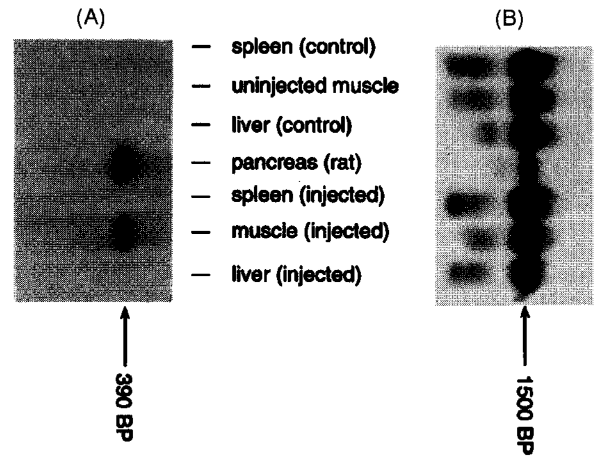

FIGURE 5 (A) Insulin RT-PCR of an experimental animal liver, spleen and muscle (lanes 5–7) showing expression of insulin only at the injection site. An experimental animal that did not respond to treatment was used as a negative control (lanes 1–3). Rat pancreas was used as a positive control (lane 4). (B) Beta-actin RT-PCR of the same samples showing intact mRNA in all samples. Representative experiment of 3 with similar results.

Animals in which the serum glc was tested were the same animals that underwent RT-PCR.

### DISCUSSION

Among the several animal models available for study of human type 1 DM, the autoimmune model is the most extensively investigated. Utilizing the NOD mouse model of type 1 DM, it was found that insulinitis resulted in destruction of pancreatic islets. Various immunomodulatory mechanisms have been able to reverse DM in NOD mice.[7] We have taken a novel approach to prevent DM in this model by supplying extra-pancreatic insulin *via* rAAV gene therapy. The basis of this approach is multifactorial. First, simple production of low levels of insulin in the prediabetic state has been shown to delay the onset of clinical diabetes.[1] Second, by producing a steady state of insulin, the opposing endocrine pathways could provide the necessary balance by the counter-regulatory system, *i.e.*, glucagon producing alpha-cells of the pancreas which are not affected by DM. Finally, while low levels of insulin may not provide complete respite from hyperglycemia, basal

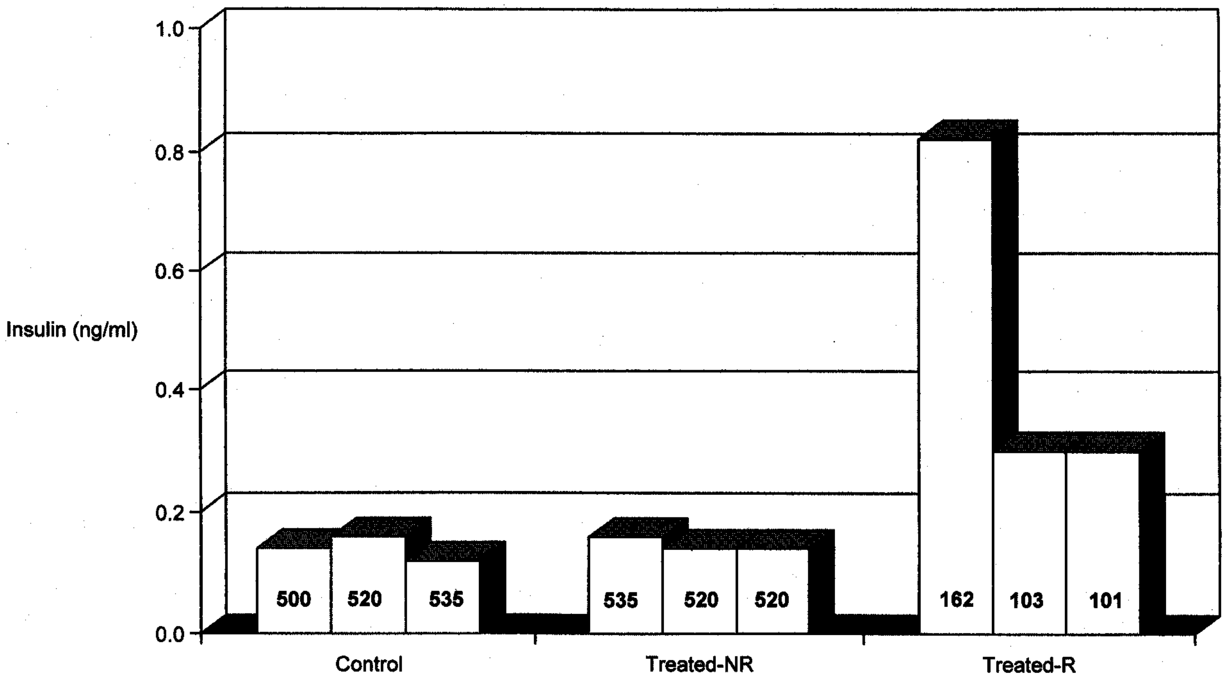

FIGURE 6 Circulating serum insulin in control diabetic, experimental animals that did not respond to treatment (Treated-NR), and experimental that responded to treatment (Treated-R) (N = 3). Numbers inside individual bars indicate serum glucose at time of sacrifice. Normal nondiabetic NOD serum insulin was 1.3 ng/ml ± 0.1.

levels would likely provide better diabetic control, and thereby, prevent the secondary complications of DM.

NOD mice develop a lymphocytic infiltrate in the pancreatic islets causing insulinitis at approximately 5–6 weeks of age and progress to overt diabetes by 12–14 weeks by an autoimmune process. It is postulated that the NOD model most closely resembles human type I DM. Previous work has shown that numerous agents have prevented DM in the NOD mouse model. In particular, mechanisms that modulate cytokine profile appeared to prevent onset of DM; IL-4, IL-10 and TGF-$\beta$ were effective.[8] Transfer of either T-suppressor cells of TGF-$\beta$ producing clones prevented DM[9] and treatment with anti-CD40 monoclonal antibody also completely prevented insulinitis.[10] Mixed allogeneic chimerism has also been found to be effective in preventing insulinitis.[11] Furthermore, experiments in pregnant NOD mice also inhibited progression of DM, as it is well known that pregnancy is an immune-privileged state.[12] Other exogenously administered agents such as Linomide[13] and certain specific peptides[14] were also effective in preventing DM in NOD mice. Finally, introduction of gene mutations and MHC Class II genes also resulted in the prevention of DM in the NOD mice.[15,16]

Another method of preventing DM is by supplying an alternative source of insulin such as by pancreatic islet transplants. Transplantation of islets utilizing semi-permeable membranes was partially successful in the prevention of DM.[17] Pretreatment of islets with various compounds also prolonged islet survival but did not impact the insulinitis.[18] The limitations of allogeneic islet cell transplantation in NOD are no different than that seen in humans, including the need of immunosuppressive agents.

In these experiments, we have provided an endogenous source of insulin by injecting the insulin gene into the skeletal muscle. Insulin released from the muscle tissues was adequate to prevent DM. We found mRNA by RT-PCR at the site of injection without obvious pathological damage to the muscle or other organs. The degree of insulinitis in NOD mice receiving vLP-1 was similar to controls despite the fact that blood glc and weight of vLP-1-injected animals was near-normal. These results suggested that vLP-1 produced insulin did not prevent the development of insulinitis. Our results are in contrast to that of Jansen *et al.*[19] who showed that prophylactic insulin treatment of NOD mice decreased insulinitis onset; H&E staining of pancreata showed decreased islet size and lower attraction of infiltrating macrophages. Experiments reported here were not intended to prevent insulinitis, but rather to provide an alternative route of endogenous insulin delivery. Likewise, since it has been reported that viruses can alter the autoimmune response, it could be argued that our animals were protected from insulinitis secondary to the viral proteins changing surface MHC expression. We discount this possibility as pancreata in both groups of animals developed an equal degree of insulinitis (reviewed blindly by a pathologist). Furthermore, there was complete absence of insulin staining in pancreas obtained from NOD mouse at week 16 (Fig. 4C) confirming the fact that there was no spontaneous reversal of insulinitis.

The presence of insulin message confined to the site of the injection in the muscle is noteworthy. Reports have shown that other genes when delivered to muscle *via* rAAV "leak" into the circulation and are detected in hepatocytes and other tissues.[20] In our experiments, other tissues were negative for the insulin gene. Furthermore, there was no histopathological damage to the muscle at the site of injection or to other mouse organs. These findings suggested that at least in the short-term there was no deleterious immunological response to the injected virus. Further doses of the virus could be delivered at regular intervals in the clinical setting.

We investigated the level of circulating insulin in the animals that were examined by histology and RT-PCR. Even though there was more circulating insulin in the experimental, non-diabetic animals, it is difficult to determine the true output of our construct. We know of no RIA that is rat insulin specific without cross-reactivity in the mouse. Furthermore, we did not look for the levels of pre-proinsulin and proinsulin. It is possible that these moieties may have also contributed to euglycemia. However, the presence of mRNA as detected by RT-PCR, and the increased amount of circulating insulin lead us to believe that our construct was responsible for the majority of insulin in the 16 week non-diabetic animals. With the currently available assays, it is not possible to say with certainty that preproinsulin encoded by the gene construct is enzymatically processed to insulin. It is known that a sizeable portion of the proinsulin that enters the immature granules of golgi network may be unprocessed and this portion may escape from the circulation. Unprocessed proinsulin has 70% cross reactivity with insulin measuring reagents. This would also explain the high plasma glucose levels accompanied by highest plasma insulin value in one of the three animals studied. Secondly, the NOD mice may have insulin receptor antibody which could perhaps explain high glucose and high insulin levels in this animal.

The question has been raised whether an insulin-specific promoter instead of RSV is necessary for gene therapy of diabetes. This issue is being explored in our laboratory. We have constructed a vector containing the preproinsulin II driven by RIP-1 to test the hypothesis that a specific promoter will result in tighter glc control. *In vitro* experiments comparing this vector against vLP-1 are in progress. Recently, Lee *et al.*[21] have shown that using rAAV that expresses a single chain insulin analogue under control of hepatocyte-specific L-type pyruvate kinase promoter was able to reverse diabetes in both STZ and NOD mice up to 5 months. In these experiments, viral particles were injected into the portal vein, a relatively invasive procedure. Our method of delivery of intra-muscular injection is simpler and more clinically relevant. However, we agree that more efficient vectors using a variety of delivery techniques will have to be investigated before a clinical trial of gene therapy for diabetes is contemplated.

We postulate, based on indirect evidence, that there was some form of autoregulation of the blood glc in the experimental animals. None of our animals showed overt hypoglycemia and had normal weight gain. It is possible that the baseline production of insulin may have been offset by increased secretion of glucagon by the alpha cells which are unaffected by insulinitis, or that the presence of glucose sensing genes in the muscle regulated the release of insulin from our construct. Further studies are planned to perform oral glucose tolerance tests and counter-regulatory hormones in animals treated with insulin gene *versus* animals not receiving the gene.

We conclude that the rAAV vector is an efficient way to mediate gene transfer. This work represents the first successful attempt at gene therapy of DM. We were able to successfully transfer the rat preproinsulin gene IM and obtain expression *in vivo* and prevent overt DM. Experiments are currently in progress to treat NOD mice which have overt DM.

### *Acknowledgements*

Mark Bochan performed some of the PCR assays and Richard A. Sidner measured some of the blood sugars. The study was funded in part by The Department of Surgery, and Darlinda's Charity, Glasgow.

### *References*

[1] Coutant, R., Carel, J. C., Timsit, J., Boitard, C. and Bougneres, P. (1997). Insulin and the prevention of insulin-dependent diabetes mellitus, *Diabetes and Metabolism*, **23**(Suppl. 3), 25–8.
[2] www.med.uni-giessen.de/itr
[3] Jindal, R. M., Sidner, R. A., Bochan, M. R. and Srivastava, A. (1998). Adeno-associated virus vectors: Potential for gene therapy, *Graft*, **1**, 147–53.
[4] Fisher, K. J., Joos, K. and Alston, J. *et al.* (1997). Recombinant adeno-associated virus for muscle directed gene therapy, *Nat. Med.*, **3**, 306–12.
[5] Peng, L., Sidner, R. A., Bochan, M. R., Burton, M. M., Cooper, S. T. and Jindal, R. M. (1997). Construction of recombinant adeno-associated virus vector containing the rat preproinsulin gene, *J. Surg. Res.*, **69**, 193.
[6] Sambrook, J., Fritsch, W. and Maniatis, T. (1989). Molecular Cloning: A Laboratory Manual, 2nd edn., New York: Cold Springs Harbor Laboratory.

[7] Leiter, E. and Serreze, D. V. (1992). Antigen presenting cells and the immunogenetics of autoimmune diabetes in NOD mice, *Reg. Immunol.*, **4**, 263–73.
[8] Tisch, R. and McDevitt, H. O. (1996). Insulin-dependent diabetes mellitus, *Cell*, **85**, 291–7.
[9] Han, H. S., Jun, H. S., Utsugi, T. and Yoon, J. W. (1996). A new type of CD+ supressor cell completely prevents spontaneous autoimmune diabetes and recurrent diabetes in syngeneic islet-transplanted NOD mice. *J. Autoimmunity*, **9**, 331–9.
[10] Balasa, B., Krahl, T. and Patstone, G. *et al.* (1997). CD40L ligand-CD40 interactions are necessary for the initiation of insulitis and diabetes in non-obese diabetic mice, *J. Immunol.*, **159**, 4620–7.
[11] Li, H., Kaufman, C. L. and Boggs, S. S. *et al.* (1996). Mixed allogeneic chimerism induced by a sublethal approach prevents autoimmune diabetes and reverses insulitis in non-obese diabetic (NOD) mice, *J. Immunology*, **156**, 380–8.
[12] Chen, H. M., Jovanovic-Peterson, L., Desai, T. A. and Peterson, C. M. (1996). Lessons learned from non-obese diabetic mouse II: Amelioration of pancreatic autoimmune isograft rejection during pregnancy, *Am. J. Perinatology*, **13**, 249–54.
[13] Slavin, S., Weiss, L., Xia, W. and Gross, D. J. (1996). Successful treatment of diabetes in NOD mice with advanced disease by islet isografts following immunoregulation with Linomide (quinoline-3-carboxamide), *Cell Transplant*, **5**, 627–30.
[14] Elias, D. and Cohen, I. R. (1995). Treatment of autoimmune diabetes and insulitis in NOD mice with heat shock protein 60 peptide p277, *Diabetes*, **44**, 1132–8.
[15] Singer, S. M., Tisch, R. and Yang, X.-D. *et al.* (1998). Prevention of diabetes in NOD mice by a mutated I-Ab transgene, *Diabetes*, **47**, 1570–7.
[16] Bohme, J., Schuhbaur, B., Kanagawa, O., Benoist, C. and Mathias, D. (1990). MHC-linked protection of diabetes dissociated from clonal deletion of T cells, *Science*, **249**, 293–5.
[17] Rivereau, A. S., Darquy, S. and Chaillous, L. *et al.* (1997). Reversal of diabetes in non-obese diabetic mice by xenografts of porcine islets entrapped in hollow fibres composed of polyacrylonitrile-sodium methallylsulphonate copolymer, *Diabetes and Metabolism*, **23**, 205–12.
[18] Fakir, M., Penfornis, A., Elian, N., Cugnenc, P. H. and Altman, J. J. (1997). Grafted immunoisolated human benign insulinoma reduces the incidence of diabetes in young NOD mice without abolishing the autoimmunity, *Int. J. Art Organs.*, **20**, 637–43.
[19] Jansen, A., Rosmalen, J. G., Homo-Delarche, F., Dardenne, M. and Drexhage, H. A. (1996). Effect of prophylactic insulin treatment on the number of ER-MP23+ macrophages in the pancreas of NOD mice. Is prevention of diabetes based on beta-cell rest? *J. Autoimmunity*, **9**, 341–8.
[20] Ponnazhagan, S., Mukherjee, P. and Yoder, M. C. *et al.* (1997). Adeno-associated virus 2-mediated gene transfer *in vivo*: organ-tropism and expression of transduced sequences in mice. *Gene*, **190**, 203–10.
[21] Lee, H. C., Kim, S. J., Kim, K. S., Shin, H. C. and Yoon, J.-W. (2000). Remission in models of type I diabetes by gene therapy using a single-chain analogue, *Nature*, **408**, 483–488.

